# A new method to determine reflex latency induced by high rate stimulation of the nervous system

**DOI:** 10.3389/fnhum.2014.00536

**Published:** 2014-07-18

**Authors:** Ilhan Karacan, Halil I. Cakar, Oguz Sebik, Gizem Yilmaz, Muharrem Cidem, Sadik Kara, Kemal S. Türker

**Affiliations:** ^1^Department of Physical Medicine and Rehabilitation, Bağcilar Training and Research HospitalIstanbul, Turkey; ^2^Institute of Biomedical Engineering, Fatih UniversityIstanbul, Turkey; ^3^Koç University School of MedicineIstanbul, Turkey

**Keywords:** human, reflex latency determination, cumulated density, EMG, averaging

## Abstract

High rate stimulations of the neuromuscular system, such as continuous whole body vibration, tonic vibration reflex and high frequency electrical stimulation, are used in the physiological research with an increasing interest. In these studies, the neuronal circuitries underlying the reflex responses remain unclear due to the problem of determining the exact reflex latencies. We present a novel “cumulated average method” to determine the reflex latency during high rate stimulation of the nervous system which was proven to be significantly more accurate than the classical method. The classical method, cumulant density analysis, reveals the relationship between the two synchronously recorded signals as a function of the lag between the signals. The comparison of new method with the classical technique and their relative accuracy was tested using a computer simulation. In the simulated signals the EMG response latency was constructed to be exactly 40 ms. The new method accurately indicated the value of the simulated reflex latency (40 ms). However, the classical method showed that the lag time between the simulated triggers and the simulated signals was 49 ms. Simulation results illustrated that the cumulated average method is a reliable and more accurate method compared with the classical method. We therefore suggest that the new cumulated average method is able to determine the high rate stimulation induced reflex latencies more accurately than the classical method.

## Introduction

High frequency mechanical and electrical stimulation of the neuromuscular system is becoming increasingly popular in scientific research (Fornari and Kohn, 2008; Nakajima et al., 2009). This is because it opens up new avenues of investigating neuronal pathways and neuromuscular performance. The whole body vibration (WBV), tonic vibration reflex (TVR) and high frequency electrical stimulation studies are examples where high frequency stimulation is used. Although the effects of such stimuli are well discussed, the response latency they generate is often ignored, as it is not observed in simple recordings (Pechstein et al., 1996; Martin and Park, 1997; Nakajima et al., 2009; Martínez-Pardo et al., 2013; Liao et al., 2014).

Conventionally, stimuli are given to the nervous system to reveal a response and the time difference between the stimulation and the response reflects the reflex latency. Usually a large number of stimuli are delivered to obtain average responses and to ensure the accuracy of the latency value. Interstimulus intervals are also kept long enough to make sure that there always is some unaffected EMG activity preceding the stimulus. Therefore, when the intervals between successive stimuli are kept long enough (>1 s), it is easy to distinguish the stimulus-evoked responses from other responses unrelated or secondary to the stimulus. If the experimental procedure requires high rate of stimuli, such as the WBV, which is the subject of this paper, the relationship between the stimulus time and the induced response becomes obscure. This is because in high rate stimulations the interval between the stimulus and the response becomes contaminated with many events such as responses from the previous stimuli and the occurrence of the subsequent stimuli (and possibly the corresponding stimulus artifacts). The high rate of WBV stimulation is faster than the reflex latency expected in a neuronal circuit and hence vibration induced reflex latency cannot be determined using the conventional manual methods.

There is another serious problem especially using WBV stimuli that involves the determination of the exact timing of the stimulus point. For single pulse electrical or very short lasting (tap-like) mechanical stimuli, it is easy to pinpoint the timing of the effective stimulus. When the stimulus duration is short, the difference between stimulus onset time and the effective stimulus time is negligible. However, the WBV stimulus is continuous and sinusoidal in nature. Consequently, the intensity of the mechanical stimulus varies periodically with time. Therefore, determination of the timing of the effective stimulus i.e., the exact location of the effective mechanical stimulus in time along one period of a vibration cycle is crucial.

Classically, solution for such problems utilized the cumulant density function method. This method is similar to the cross-correlation histograms and the spike-triggered averaging methods. It objectively reveals the relationship between the two synchronously recorded signals as a function of the lag between the signals (Halliday and Rosenberg, 1999). However, the analysis does not provide a solution to determining the effective stimulus point and the response onset point. This is because, although the cumulant density method uses effectively the peaks of signals to estimate the lag time, this method does not attempt to indicate the exact time of the effective stimulus and the time of the initial response in the signal.

Thinking that each vibration stimulus, regardless its rate, generates a reflex response on the EMG at a fixed latency, we predicted that it is possible to calculate WBV induced reflex latency using an averaging method. To achieve this goal, we determined the timing of the effective stimulus and the onset of the reflex EMG activity by using “cumulated average method”. We averaged the data obtained during all the six vibration frequencies tested in this study. We then combined all the averaged data belonging to six different vibration frequencies to generate the ***cumulated average*** for each participant. We also obtained the standard error (SE) for the cumulated average records. Lowest value of the SE indicated timing of the effective stimulus and the onset of the reflex response. Our principal hypothesis was that the ***cumulated average*** method is capable of determining the time of the effective stimulus and the onset of the reflex response in addition to determining of the peak-to-peak latency. A computer simulation was performed in order to test reliability of our method in a simulated condition where the response latency was known. This simulation study also compared our method with that of the cumulant density function method for accuracy.

## Methods

### Participants

Five healthy volunteers (4 females, 1 male; mean ± SD: age 24.2 ± 1.3 years; height 168.0 ± 7.1 cm; body mass index 21.3 ± 1.1 kg/m^2^) participated in the study. All participants gave written informed consent to the experimental procedures, which were in accordance with the Declaration of Helsinki and were approved by the local ethics committee (Istanbul University Istanbul Medical Faculty Clinical Research Evaluation Committee, Istanbul; July 5, 2013/13).

### Procedures

During WBV, the participants were asked to stand upright on a vibration platform (POWERPLATE® Pro5, London, United Kingdom). Their hips and knees were in a neutral position. Participants were barefooted, and no sponge or foam was placed between the vibration platform and their feet. The whole plate oscillated with a linear movement upward and downward. Vibration amplitude was 2.2 mm. Accelerations for the WBV frequencies of 25, 30, 35, 40, 45 and 50 Hz varied between 4.0 and −2.0 g, 4.4 and −2.9 g, 4.4 and −4.1 g, 4.4 and −4.6 g, 4.4 and −4.7 g and, 4.4 and −4.7 g, respectively. WBV frequencies were varied randomly to cover all six frequencies. Each frequency lasted for 60 s and there was a 15 s rest in between different frequencies.

A load sensor (FC2331-0000-2000L Compression Load Sensor, France) was fixed on the WBV platform in order to be able to record the vibration-induced force data. Participants were positioned on the WBV platform such that their right heel was placed on the load sensor.

### Recordings

Multi motor unit (MMU), surface electromyography (SEMG) and force data were recorded simultaneously using the POWERLAB® data acquisition system (ADInstruments, Oxford, United Kingdom). A sampling frequency of 40 kHz was used. Data were analyzed offline using the MATLAB software (R2012a 7.14.0.739). In order to record SEMG, Ag/AgCl surface electrodes (KENDALL® Arbo) with a disc radius of 10 mm were placed 20 mm apart on the right soleus muscle belly, on shaved skin cleaned with alcohol.

To record MMU activity, a pair of TEFLON® insulated silver wires (75 μm in core diameter) was used. About 3 mm of the tips of the silver wires were stripped off their TEFLON® coating in order to be able to record the activity of several motor units. The pair of wires was inserted into the right soleus muscle with a 25 G needle. Needle was immediately withdrawn leaving the fish-hooked wires in the muscle electrode (for details of the methods please refer to Brinkworth and Türker, 2003). A lip-clip electrode was used as the ground (Türker et al., 1988).

All wires were carefully taped to the skin to minimize mechanical artifacts. The participants were instructed to relax their muscles throughout the recordings and were trained using EMG feedback to this end. WBV may impair the sense of balance and muscles may be activated to restore balance. To overcome this problem, the participants were familiarized with WBV with a 15 s trial session on the WBV device and they were asked to use the handles of the device to secure their balance. The distance between the heels was about 25 cm to further stabilize their balance.

### Filtering and rectification regimens

All records were processed with infinite impulse response (IIR) filters (Butterworth, 1st order) using MATLAB (R2012a 7.14.0.739). All force sensor recordings were 5 Hz high-pass filtered. All SEMG recordings were 80–500 Hz band-pass filtered and full-wave rectified to overcome movement artifacts (Sebik et al., 2013). All MMU recordings were 100–5000 Hz band-pass filtered and full-wave rectified.

### Data analyses

Data were analyzed using both the cumulated average method and the cumulant density function.

#### Cumulated average method

The time between the effective stimulus point and the onset of the reflex EMG (MMU or SEMG) activity was defined as the WBV induced reflex latency in this study. The effective stimulus point and the onset of the reflex EMG activity were determined as follows:

#### Determination of the timing of the effective stimulus

**Figure 1:**
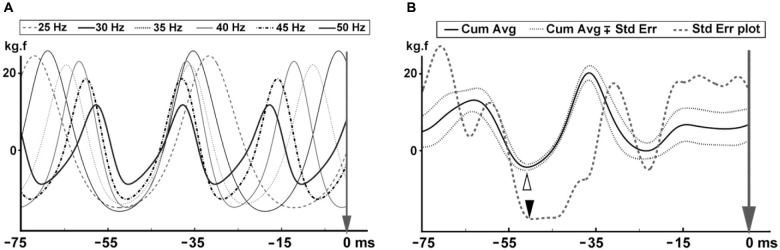
**Determination of the position of the effective stimulus in the force trace for one participant. (A)** Averaged force data for each of the six different vibration frequencies. The vertical arrow shows the peak of MUAP that was used as the trigger for averaging the force data. **(B)** Mean (thick line) and standard error (SE) (dotted lines around the mean trace) for the cumulated average force trace. Thick continuous line shows the mean of the cumulated average force with its minimum value indicated using the white arrowhead. Thick dashed line is the SE for the cumulated average force trace with its minimum value indicated using the black arrowhead.

The first and last 5 s of the force data were discarded to eliminate vibration onset and finish effects, and the remaining 50 s were further analyzed. The peaks in time derivatives of rectified MMU and SEMG traces were marked using MATLAB (R2012a 7.14.0.739) software. Peaks of the time derivative of rectified EMG were used as triggers to average the force data between the time of the trigger and 75 ms segment preceding the trigger. This averaging process was separately conducted for the six WBV sets, each of which used different vibration frequencies. Number of such triggers ranged between 1245 and 2490 depending on the vibration frequency. Averaged force data belonging to six different vibration frequencies were then combined to generate the ***cumulated average*** of the force for each participant. The standard error (SE) values for each data point on the cumulated average force records were also calculated. The point in time in a force cycle where the SE was the lowest was considered as the effective stimulus time point (Figure 1).

#### Determination of the onset of the reflex EMG activity

**Figure 2:**
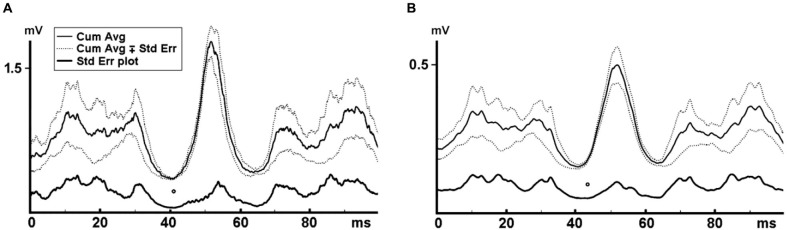
**Cumulated average MMU and SEMG where the local minimum of the force trace was used as the trigger**. Reflex latency was indicated by the lowest SE on the cumulative averaged data and shown with an empty circle. **(A)** Indicates the MMU data and **(B)** shows the SEMG data. Reflex latency for the MMU data was 38.0 ms and 40.0 ms for the SEMG data.

Similar to the approach in the force data, the first and last 5 s of the EMG data were discarded to eliminate vibration onset and finish effects, and the remaining 50 s were further analyzed. The effective stimulus points were marked for each vibration cycle in the force trace. Rectified MMU and SEMG traces were averaged covering the 100 ms from each time point of the effective stimulus point. This process was separately conducted for each of the six WBV sets with different vibration frequencies. Number of triggers ranged between 1245 and 2490 depending on the vibration frequency. All the averaged rectified MMU and SEMG data belonging to six different vibration frequencies were then combined to generate the cumulated average of the MMU and SEMG for each volunteer. The SE values for each data point on the cumulative averaged records were calculated. Lowest SE on the cumulated average of the EMG data was considered as the time point where the reflex responses to the stimuli were most synchronized and hence the onset of reflex response (Figure 2).

#### Cumulant density function analysis

This method is classically used as the gold standard for similar experiments. This method uses peaks of signals to determine a lag time between two synchronously recorded signals. Cumulant density function analyses were conducted for both Force-SEMG and Force-MMU pairs. Full wave rectification was used to maximize information about action potential timing. In the frequency domain, estimates of the auto spectrum of the force and EMG were constructed, along with estimates of coherence. Timing information between the force and EMG signals was calculated from the phase spectrum. In the time domain, the cumulant density function, with EMG as reference, was estimated from the cross spectrum, via an inverse Fourier transform. Confidence intervals for all parameters were estimated (Halliday et al., 1995). Cumulant density analyses were conducted using the Neurospec (version 2.0) code available for MATLAB (Halliday, 2008).

### Simulation

A simple simulation protocol was used to test the accuracy of our method in a simulated condition where the reflex response latency is predetermined, and to compare the method with the cumulant density function method. Sixty seconds of SEMG and force data at a sampling rate of 10 kHz for each vibration frequency (25, 30, 35, 40, 45 and 50 Hz) were simulated. SEMG representations of 10 different motor unit action potentials (MUAPs) were extracted from genuine SEMG recordings and added randomly at the predetermined latency, with regards to the selected effective stimulus point in the simulated force data, in order to form the simulated SEMG signal. Time difference between the effective stimulus point and onset of EMG response (reflex latency) was selected as 40 ms. Force data were generated as pure sines with the six frequencies of 25, 30, 35, 40, 45 and 50 Hz. Peak to peak amplitude of force was divided into four equal parts in the simulated force traces with, the first quartile starting with the negative peak. The point between the first quartile and second quartile was designated as the effective stimulus point. Then, the exact protocol as in our genuine EMG/force data was followed on the simulated data to examine which of the two methods was more accurate in pinpointing the predetermined “reflex” latency.

### Statistical analysis

To determine confidence interval (CI) of mean, mean and SE were calculated for all data. When comparing means of two data sets, if 95% CI of one data set included mean of other data set, it was accepted that there is no statistical difference between the means of two data sets. The Wilcoxon test was used to analyze the statistical difference between the mean reflex latency and mean lag time. The data management software package used was PASW® for Windows.

## Results

### Reflex latency

In the new technique, we first determined the position of the effective stimulus so that it could be used as the stimulus onset point for further analyses. As described in the Methods section, we used the peaks in the time derivatives of SEMG and MMU traces as the trigger and force trace as the source to perform a spike triggered averaging (STA) process for all the six WBV frequencies tested. We then combined the STA data for all six frequencies to observe the lowest SE on the cumulated average force data. Figures 1A,B illustrates the method used to determine the timing for the effective mechanical stimulus.

**Figure 3:**
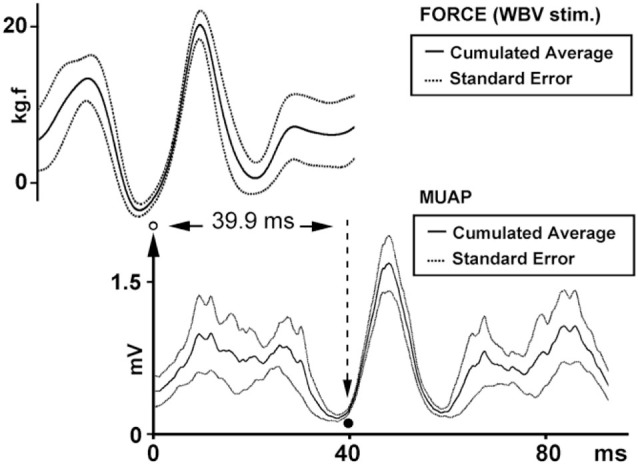
**Reflex latency measurement using cumulated average method.** The arrow and open circle shows the position of the effective stimulus point (the lowest value of the SE of force trace). Solid circle shows the onset of EMG response (the lowest value of the SE of MMU trace).

Figure 2 illustrates a cumulative averaged rectified SEMG and MMU traces and their SE values for one participant. When the effective stimulus points of the force trace were taken as the trigger and the rectified EMG responses to all six WBV frequencies were used as the source in a STA process, we noted that the lowest SE value for the cumulative averaged rectified SEMG and MMU traces were about 32 ms for all the participants (Table 1). Figure 3 shows measurement of reflex latency using cumulated average method.

**Table 1:** **The reflex response latency calculated with the cumulated average method**.

Participant (Gender)	Latency (ms) (MMU data)	Latency (ms) (SEMG data)
1 (Female)	33.7	33.9
2 (Female)	37.8	38.5
3 (Female)	25.7	28.2
4 (Male)	38.0	39.9
5 (Female)	22.3	23.6
Mean ± SE	31.5 ± 3.2	32.2 ± 2.1
95% CI	25.2–37.8	26.8–39.0

**Figure 4:**
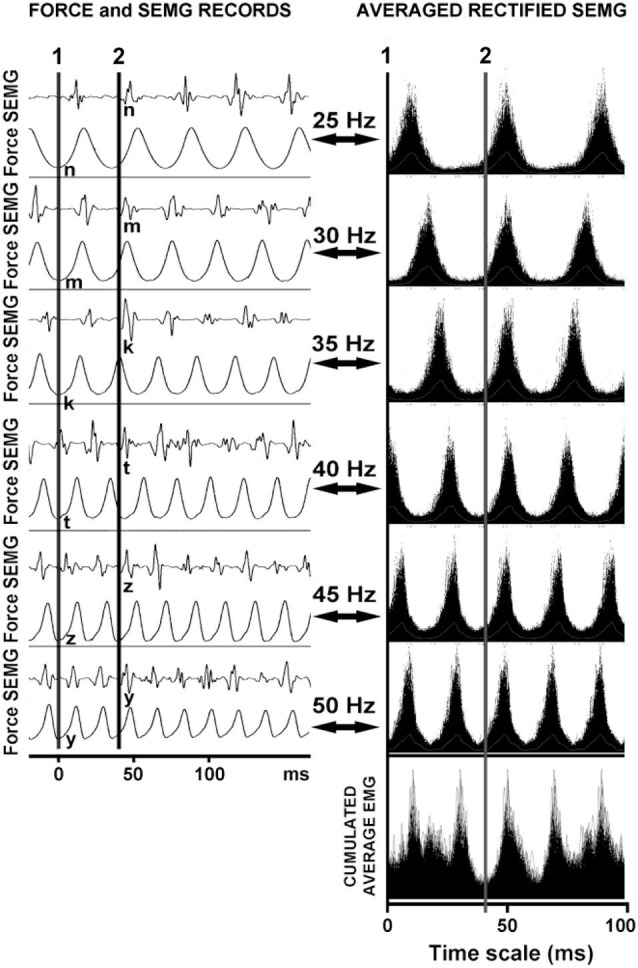
**The reflex latencies for different frequencies of WBV and the logic underlying the cumulated average method.** The line 1 represents the position of the effective stimulus point and the line 2 represents the reflex latency of the system to that stimulus. Note that the reflex responses are lined up even though the WBV frequencies were different.

Figure 4 shows that each vibration stimulus, regardless the vibration frequency, generates a reflex response at the same latency.

### Peak to peak latency (Lag time)

**Figure 5:**
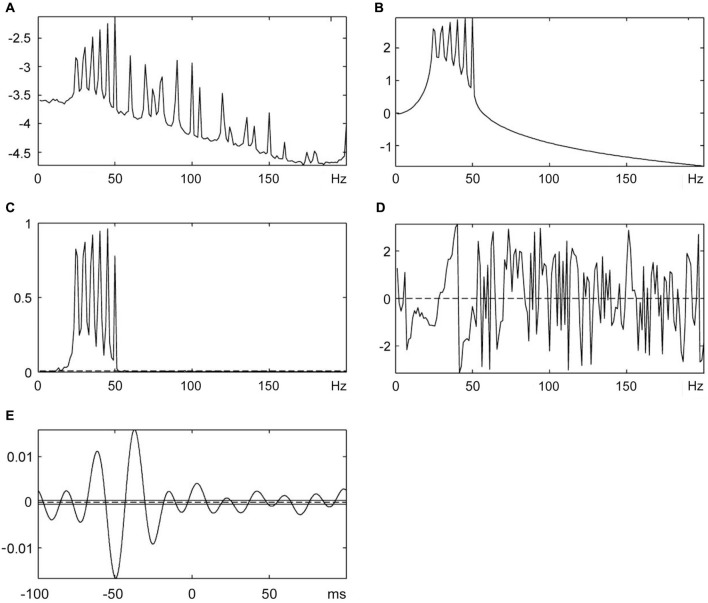
**The cumulant density analysis for Force-MMU data. (A)** Spectral analysis of SEMG data, **(B)** Spectral analysis of force (vibration stimuli), **(C)** The coherence analysis shows a significant correlation between SEMG and vibration signals, **(D)** Phase analysis and **(E)** Lag time between SEMG and vibration stimuli.

The cumulant density analysis showed a significant relationship between Force-MMU signals and Force-SEMG signals in all the individuals (Figure 5). The lag time between force (vibration stimuli) and the related EMG responses calculated by cumulant density function was given in Table 2. There was no statistically significant difference between peak to peak latency obtained using the cumulated average method and the lag time obtained using the cumulant density calculations. However, the reflex latency, as determined using the current method, was significantly shorter than the lag time obtained using the cumulant density calculations (*p* < 0.05 for both SEMG and MMU data).

**Table 2:** **Peak to peak (PP) latency between the force and EMG signals calculated with cumulated average method and the lag time between the force and EMG signals calculated with cumulant density function**.

Participant (Gender)	PP Latency (ms) (MMU data)	PP Latency (ms) (SEMG data)	Lag time (ms) (MMU data)	Lag time (ms) (SEMG data)
1 (Female)	49.3	49.4	48.7	47.3
2 (Female)	50.4	50.5	50.4	49.1
3 (Female)	48.9	49.4	47.8	48.4
4 (Male)	50.5	51.0	50.6	50.0
5 (Female)	46.2	45.1	45.1	43.5
Mean ± SE	49.0 ± 0.8	49.1 ± 1.1	48.5 ± 1.0	47.7 ± 1.1
95% CI	47.5–50.6	47.0–51.1	46.5–50.5	45.5–49.9

### Simulation findings

**Figure 6:**
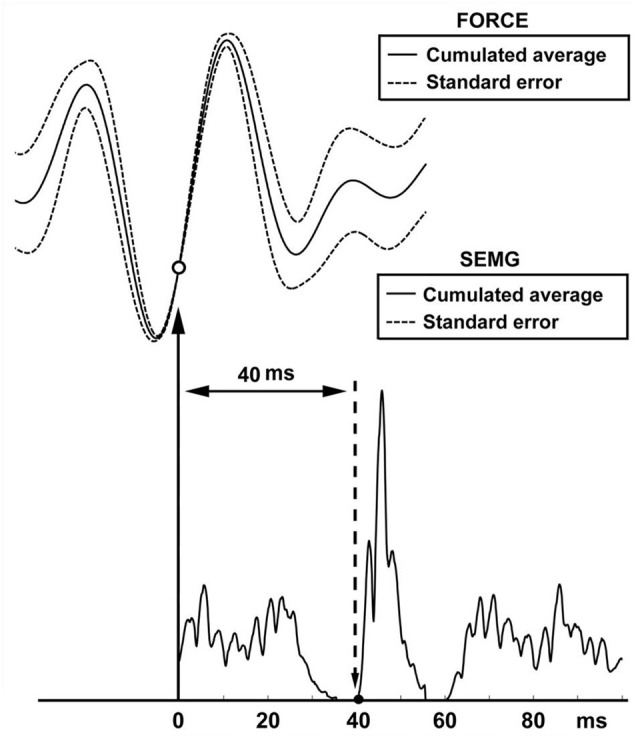
**Reflex latency measurement using cumulated average method in simulation data**. Open circle shows the lowest value of the SE of force trace (effective stimulus point). Solid circle shows the lowest value of the SE of EMG trace (onset of EMG response).

**Figure 7:**
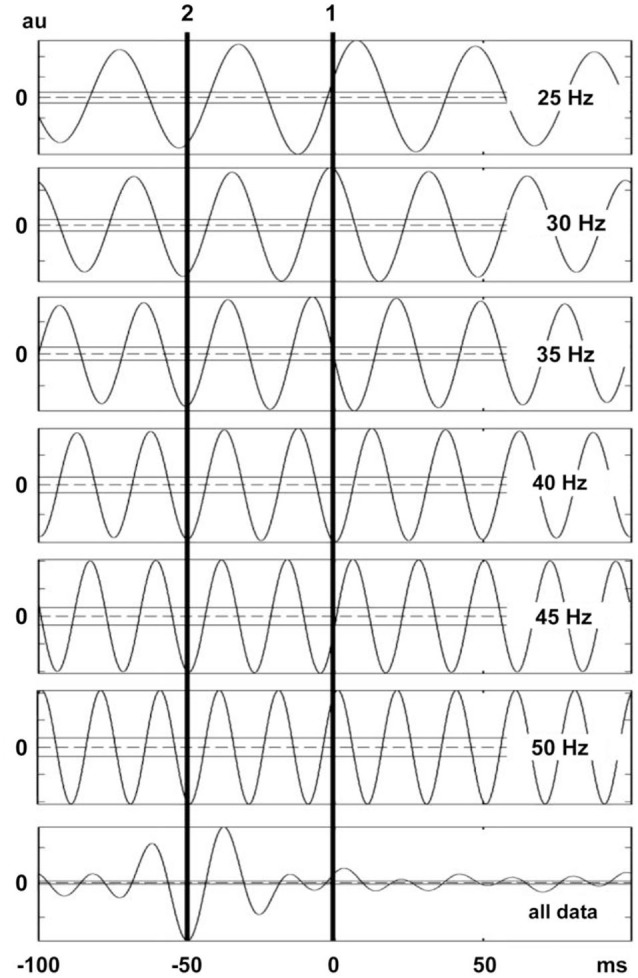
**Lag time between vibration stimuli and EMG response.** This figure shows the lag time calculated by using cumulant density analysis for six vibration frequency. The second vertical line corresponds to the lag between the local minima of the force trace and the peaks in the rectified MUAP. Note that the lag time between local minima in force trace and the related MUAP peaks are lined up even though the WBV frequencies were different. The confidence limits are marked with horizontal lines in the graphs.

The findings of the simulated SEMG signals were in agreement with the findings of the SEMG recordings. In simulated signals where the reflex latency was constructed as 40 ms, reflex latency determined using the cumulative average method was 40 ms (Figure 6). However, the lag time between force and EMG signals calculated using the cumulant density function method was 49 ms (Figure 7).

## Discussion

In support with our hypothesis, the following original results were found: firstly, the latency of the reflex response to the WBV can be determined using the new method described. Secondly, this method is superior the cumulant density analysis as it takes into account the timing of the effective stimulus and the initial response of the muscle. Finally, the computer simulation study confirmed the accuracy of our findings.

### Latency measurements

Even though reflex latency measurements could be problematic, it is not difficult to illustrate a relationship between two signals if one of the signals triggers the emergence of the other signal (stimulus and response signal matching). Since an EMG spike appears for each vibration cycle during WBV vibration, the force recording can be accepted as the stimulus signal and EMG spike recordings as the response signal (Figure 4). This matching indicates that there is a specific relation between vibration stimulus and EMG spike in time.

Ritzmann et al. (2013) hypothesized that the latency during WBV can be calculated using the point in time where the vibration platform is at the lowest position to the onset of the first spike in the EMG signal. However they have not shown that the timing of the vibration stimulus coincided with the lowest position of the vibration platform. More importantly, it is also not convenient to use the first EMG spike for the latency measurements. If the first EMG spike emerging after a vibration stimulus is used for latency measurements, the latency would vary depending on the frequency of vibration (Figure 4).

Our method relies on the fact that each stimulus, regardless the interstimulus interval, generates a reflex response with the same latency. In other words, when the stimulus pulses delivered at varying rates are superimposed, the reflex responses that correspond to each stimulus overlap at a time point that indicates the response latency (Figure 4). We used this approach to determine the reflex response of the human soleus muscle to WBV. The time between the effective stimulus point and onset the EMG response was calculated as WBV induced reflex latency.

For this, we had the following assumptions:

- During WBV, vertical vibration stimulus “stretches” the leg muscles at a certain phase of the vibration;
- This stretch stimulus activates the available muscle spindles and hence induces all postural muscles of the leg to contract;
- Since stretch is the stimulus for the spindles of the leg muscles, regardless the vibration frequency, the reflex response will have the same delay from the time of the adequate stimulus;
- Therefore, if we superimpose EMG responses that are obtained at different WBV frequencies they will be synchronized at the same reflex latency.

The above assumptions seem to be justified in this study since as shown in Figure 4 all synchoronous EMG responses were lined up against a certain phase of the force record for all vibration frequencies used. We therefore used this same approach in our simulation work and placed the EMG response at a fixed time period after a certain phase of the force record.

### Comparing the classical method and the new method

The cumulant density analysis is a powerful and widely used method for determining a correlation between synchronously recorded two signals. The cumulant density analysis is defined by the inverse Fourier transform of the cross-spectrum. Existence of a relationship between simultaneously recorded signals is revealed with covariance analysis and cross-spectral analysis. The coherence analysis shows the existence of correlation between the two signals and its magnitude. The lag time between two signals is obtained in time domain with inverse Fourier transform. The existence of a relationship between two signals, its magnitude and lag time within the 95% of confidence limit is then estimated. Most of the signals are stochastic and include noise. The relation between two signals is affected by the presence of noise. The confidence limits for showing a significant correlation between two stochastic signals are estimated by taking the noise into account. Thus, it is possible to distinguish the rhythmic components of the spectrum from the random fluctuations (Halliday and Rosenberg, 1999).

The cumulant density analysis showed that there was a significant relationship in time and frequency domain between the force signal and SEMG signal in this study (Figure 5). It was found that the lag time between force (vibration) stimuli and the related EMG response was around 48 ms and was not statistically different from peak to peak latency measured by cumulated average method. The reflex latency as determined from the effective stimulus point to the onset of the EMG spike was significantly different from the lag time obtained using the cumulant density method (Tables 1, 2). However, even though the cumulant density method can estimate lag time using the peaks of the events, it cannot determine the timing of the onset of the EMG and the timing of the effective force. Therefore, it cannot determine the reflex latency. This property is a major advantage of our method over and above the cumulant density method.

### Simulation study

Simulation was especially useful to determine reliability of our method for measuring vibration-induced reflex latency. In our simulation scenario, the point between the first quartile and second quartile was designated as effective stimulus point. Time difference between the effective stimulus point and onset of EMG response (reflex latency) was designated as 40 ms. In this experimental condition, cumulated average method showed reflex latency as 40 ms (Figure 6). However, cumulant density analysis showed that lag time between vibration and SEMG simulated signals was 49 ms (Figure 7). This simulation study showed that cumulated average method is a reliable method and more accurate than cumulant density analysis method for measuring vibration-induced reflex latency.

## Conclusions and clinical application

This study showed that the new cumulated average method can indicate stimulus induced response latencies even if interstimulus interval is shorter than the latency of a response. We used WBV as an example to illustrate this point. Similar to other forms of high rate stimulations (Pechstein et al., 1996; Fornari and Kohn, 2008), WBV is used in clinical and physiological research with an increasing interest. WBV induces reflex response in muscles, but the physiological mechanism underlying this reflex activity remains unclear (Pollock et al., 2012). Latency measurements of muscular reflex that is triggered by WBV may be crucial in order to reveal the underlying physiological mechanisms. The new method could pave new ground to investigate the reflex pathways activated by/during various high rate stimuli, and make it possible to compare the reflex latencies thus measured to latencies measured for known reflex pathways under different experimental conditions (Ribot-Ciscar et al., 1998; Wilcock et al., 2009; Rittweger, 2010; Pollock et al., 2012).

## Conflict of interest statement

The authors declare that the research was conducted in the absence of any commercial or financial relationships that could be construed as a potential conflict of interest.
